# A literature search of the top 100 cited papers in cutaneous lupus erythematosus

**DOI:** 10.1111/srt.13894

**Published:** 2024-08-03

**Authors:** Isabella J. Tan, Bianca Sanabria, Aarushi K. Parikh, Babar Rao

**Affiliations:** ^1^ Rutgers Robert Wood Johnson Medical School The State University of New Jersey Piscataway New Jersey USA; ^2^ Center for Dermatology Rutgers Robert Wood Johnson Medical School Somerset New Jersey USA; ^3^ Department of Dermatology Weill Cornell Medicine New York New York USA

To the Editor,

Cutaneous lupus erythematosus (CLE) represents a subset of lupus erythematosus (LE) characterized by diverse autoimmune‐mediated skin manifestations.^1^ These include discoid lupus erythematosus (DLE), presenting with erythematous plaques and scarring; subacute cutaneous lupus erythematosus (SCLE), featuring photosensitive annular lesions; acute cutaneous lupus erythematosus (ACLE), typified by transient malar rash; and chronic cutaneous lupus erythematosus (CCLE), including persistent plaques with scarring.^2^

The pathogenesis involves genetic predisposition, environmental triggers including UV light, and dysregulated immune responses with autoantibody production (e.g., anti‐Ro/SSA).^2^ Diagnostic advancements include immunofluorescence microscopy for immune complex detection in skin biopsies and serological tests for specific autoantibodies. Despite progress, challenges remain in epidemiology, classification, and treatment.

Bibliometric analyses offer valuable insights into the trends within this field.^3^ Our study analyzes the top 100 cited papers in CLE, identifying influential contributions, research themes, and evolving trends.

We conducted a comprehensive search for the top 100 most cited research articles on cutaneous lupus from January 2000 to December 2023 using Web of Science, targeting full‐text articles published in peer‐reviewed dermatology journals. On April 23, 2024, our search yielded 5750 articles. Two independent researchers reviewed the retrieved data and the top 100 most cited articles were categorized into basic science research, clinical research, or new treatment categories.

The majority (67%) of the top 100 cited articles in cutaneous lupus research were clinical research articles (Figure 1). Descriptive statistics for these seminal studies showed a mean Weighted Score (WoScore) of 85.76 (SD = 48.34), indicating moderate to high impact on research and clinical practice. Additionally, these articles had a mean citation count of 92.03 (SD = 53.01) and a mean journal impact factor (IF) of 7.19 (SD = 5.42), underscoring their influential stature.

**FIGURE 1:**
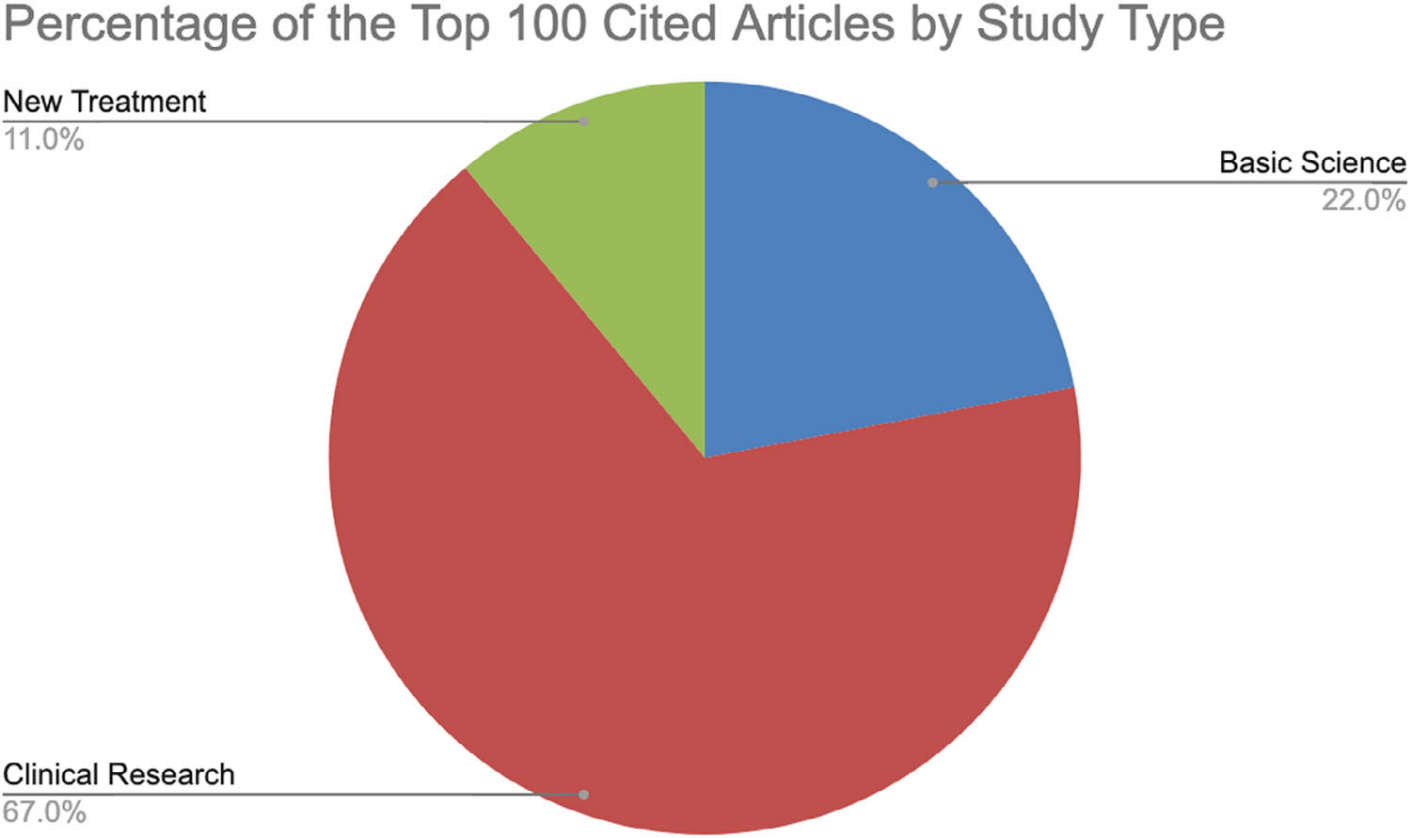
Percentage distribution of article types among the top 100 cited articles.

Examining journal‐specific data reveals distinct patterns: the *British Journal of Dermatology*, with its mean WoScore of 76.64 (SD = 30.78) and mean citation count of 80.71 (SD = 32.67) across 28 articles (Table 1). Similarly, the *Journal of Investigative Dermatology* and *Archives of Dermatology* demonstrate high mean WoScores (123.9 and 89.42, respectively) and citation counts (134.6 and 94.58, respectively, both with higher standard deviations), indicating significant contributions to advancing knowledge in cutaneous lupus. Themes in these studies collectively advanced understanding across clinical, pathogenic, therapeutic, and epidemiological perspectives, and include various facets: diagnosis using tools including CLASI (cutaneous lupus erythematosus disease area and severity index), treatment strategies (e.g., responses to antimalarials and indications for thalidomide), pathogenesis centered on interferon and cytokine pathways, exploration of CLE subtypes such as tumid lupus erythematosus and drug‐induced variants, epidemiological studies on incidence and prevalence, and considerations of quality of life and patient outcomes.

**TABLE 1:** Journal distribution of T100 articles in cutaneous lupus erythematosus.

Rank	Journal name	No. of articles
1	*British Journal of Dermatology*	28
2	*Journal of The American Academy of Dermatology*	14
3	*Archives of Dermatology*	12
4	*Journal of Investigative Dermatology*	12
5	*Archives of Dermatological Research*	5
6	*American Journal of Clinical Dermatology*	4
7	*Journal of The European Academy of Dermatology and Venereology*	4
8	*JAMA Dermatology*	2
9	*Clinics in Dermatology*	2
10	*International Journal of Dermatology*	2
11	*European Journal of Dermatology*	2
12	*Experimental Dermatology*	2
13	*American Journal of Dermatopathology*	2
14	*Dermatology*	2
15	*Dermatologic Therapy*	1
16	*Pediatric Dermatology*	1
17	*Journal der Deutschen Dermatologischen Gesellschaft*	1
18	*Journal of Cutaneous Pathology*	1
19	*Photodermatology Photoimmunology & Photomedicine*	1
20	*Dermatologic Clinics*	1
21	*Dermatology and Therapy*	1

These findings reveal a dynamic field of CLE research, marked by ongoing clinical advancements and emerging therapies. The distribution of articles over 2 decades underscores the sustained interest in understanding CLE's pathogenesis, epidemiology, and management, informing researchers, clinicians, and funders about critical areas and future directions in autoimmune skin disorders.

## CONFLICT OF INTEREST STATEMENT

Dr. Rao is a speaker for Incyte. All other authors have no disclosures.

## Data Availability

The data that support the findings of this study are available from the corresponding author upon reasonable request.
